# Mesoporous Tungsten Trioxide Photoanodes Modified with Nitrogen-Doped Carbon Quantum Dots for Enhanced Oxygen Evolution Photo-Reaction

**DOI:** 10.3390/nano9101502

**Published:** 2019-10-22

**Authors:** Mabrook S. Amer, Prabhakarn Arunachalam, Abdullah M. Al-Mayouf, Saradh Prasad, Matar N. Alshalwi, Mohamed A. Ghanem

**Affiliations:** 1 Electrochemical Sciences Research Chair (ESRC), Chemistry Department, College of Science, King Saud University, Riyadh 11451, Saudi Arabia; msamer@ksu.edu.sa (M.S.A.); parunachalam@KSU.EDU.SA (P.A.); amayouf@ksu.edu.sa (A.M.A.-M.); malshalwi@KSU.EDU.SA (M.N.A.); 2 Department of Astronomy and Physics, College of Science, King Saud University, Riyadh 11451, Saudi Arabia; sprasad@KSU.EDU.SA

**Keywords:** *meso*porous, tungsten, trioxide, carbon, quantum, dots, photo-electrochemical, water splitting

## Abstract

Nanostructured photoanodes are attractive materials for hydrogen production via water photo-electrolysis process. This study focused on the incorporation of carbon quantum dots doped with nitrogen as a photosensitizer into *meso*porous tungsten trioxide photoanodes (N-CQD/*meso*-WO_3_) using a surfactant self-assembly template approach. The crystal structure, composition, and morphology of pure and N-CQD- modified *meso*porous WO_3_ photoanodes were investigated using scanning electron and transmission microscopy, X-ray diffraction, and X-ray photoelectron spectroscopy. Due to their high surface area, enhanced optical absorption, and charge-carrier separation and transfer, the resulting N-CQD/*meso*-WO_3_ photoanodes exhibited a significantly enhanced photocurrent density of 1.45 mA cm^−2^ at 1.23 V vs. RHE under AM 1.5 G illumination in 0.5 M Na_2_SO_4_ without any co-catalysts or sacrificial reagent, which was about 2.23 times greater than its corresponding pure *meso*-WO_3_. Moreover, the oxygen evolution onset potential of the N-CQD/*meso*-WO_3_ photoanodes exhibited a negative shift of 95 mV, signifying that both the charge-carrier separation and transfer processes were promoted.

## 1. Introduction 

Photo-electrochemical (PEC) water splitting is a sustainable technology for the production of hydrogen fuel using water as the hydrogen source [1,2]. During the last decade, semiconductor metal oxide (SMO) photoelectrodes such as Fe_2_O_3_ [3], TiO_2_ [4], ZnO [5], and BiVO_4_ [6] have received considerable interest as favorable catalysts for converting solar energy into hydrogen fuel. Among them, tungsten trioxide (WO_3_) is recognized as a favorable candidate for visible-light assisted water splitting due to its moderately narrow band energy gap (Eg = 2.6–2.8 eV), highly positive valence band energy levels for PEC water oxidation (3.0 V vs. RHE), and good stability against photo-corrosion [7,8]. SMO nanostructures with *meso*porous architectures are favorable for application as solar water splitting photoanodes because of their distinctive chemical and physical features [8,9]. In particular, their huge specific surface area delivers a higher density of available active sites as well as a greater light absorption depth, both of which lead to improved photocatalytic performance. The syntheses of ordered *meso*porous materials are classified as template-free or templating methods. Methods involving sol-gel [10], chemical vapor deposition (CVD) [11], and spray pyrolysis deposition [12] mostly produce materials with irregular *meso*porous structures and disordered pore morphology. Besides, the hard-templating method produces organized *meso*porous materials with highly crystalline framework that replicates the structure of the *meso*porous SBA−15 silica, carbon, or cubic KIT-6 silica template [13,14]. However, this method has the disadvantages of being expensive and time-consuming and requires complex procedures. Therefore, it is not suitable for mass production applications. Synthetic methods using soft templates based on amphiphilic block copolymers or surfactants are another efficient and useful option for the preparation of *meso*structured materials with various morphologies and tunable pore architectures and can be used in large-scale production [15,16]. Triblock copolymer templates, such as Pluronic^®^ F−127 and Pluronic^®^ P123, are commonly employed for the fabrication of *meso*porous materials [17,18]. Unfortunately, these templates have a short segment chain length and low glass transition temperature (Tg), which results in the creation of *meso*porous materials with low crystallinity, amorphous or semi-crystalline channel walls, and uncontrollable pore size, which are not beneficial for solar water splitting devices [19,20,21].

Consequently, amphiphilic high molecular weight di-block copolymers, such as polyethylene oxide-b-polystyrene (PEO-b-PS), poly(methyl methacrylate) (PMMA), and poly(2-vinyl pyridine)-b-polystyrene (P2VP-b-PS), have recently been employed for the synthesis of *meso*porous SMOs [22,23]. In contrast to triblock copolymers, the di-block PEO-b-PS copolymer has the unique properties of strong hydrophobic/hydrophilic segments, a higher glass transition temperature, and abundant sp^2^ hybridized carbon in the PS segments, which can be in situ converted into rigid amorphous carbon during the crystallization of the framework in an inert atmosphere. Thus, its ordered *meso*structured is retained when it is used as a template [23,24]. Moreover, to advance the PEC response of electrodes for water splitting applications, the precious metals loading Ag, Au, Pt, and Pd [25,26,27], as well as photosensitizers such as CdS [28], Bi2S3 [29], and CdSe [30], have been widely employed to modify SMO photoanode materials. However, these photosensitizer materials are unstable and are easily oxidized under the operating conditions of PEC anodes, which limit their potential usage in large-scale applications [31]. In contrast, the incorporation of carbon quantum dots (CQDs) and nitrogen-doped CQDs (N-CQDs) into SMO materials as a photosensitizer is considered to be promising, as carbon quantum dots are cost-effective, electrochemically stable, non-toxic, and composed of earth-abundant elements [32]. The CQDs can act as electron donors and acceptors, leading to the creation of hole/electron pairs and thus enhancing the light absorption and photocatalytic performance of the CQDs/SMO composites. Several reports of the incorporation of a CQD photosensitizer into SMOs to produce high-performance photocatalytic photoanodes have been published recently [33,34,35]. However, CQDs still have low photosensitization efficiency, which limits their utility for advanced practical applications. More recently, Lim and coworkers demonstrated that doping CQDs with heteroatoms such as nitrogen could significantly increase their light-harvesting, tuning band structure, and optical properties, and thus greatly enhance the PEC properties of hybrids, including the doped CQDs [36]. Zhao et al. demonstrated a facial self-assembly route to integrate ultra-small graphitic N-doped pencil nanodots (PND) into a highly ordered TiO_2_ *meso*porous framework [37]. The hybrid catalyst showed high PEC activity and achieved a photocurrent density of ∼1.73 mA cm^−2^ at 0.23 V vs. Ag/AgCl, which was 183 and 108% higher than that of the pure TiO_2_ and undoped PND-TiO_2_, respectively. The PEC performance was credited to the enhanced charge transfer rate between the TiO_2_ frameworks and the modified PNDs through electron donation by the N atoms. Within this context, hybrid materials combining *meso*porous tungsten trioxide (*meso*-WO_3_) and CQDs or N-CQDs have never been reported and could be a favorable candidate for promoting PEC performance for water oxidation. Herein, we reported a facile synthetic route for the fabrication of ordered *meso*-WO_3_ via a dip-coating method and evaporation-induced self-assembly (EISA) using tetrahydrofuran (THF) as the solvent and PEO-b-PS as a template, followed by thermal treatment. The produced *meso*-WO_3_ thin films were then decorated with various loadings of CQDs and N-CQDs via an impregnation method. The N-CQD/*meso*-WO_3_ hybrid materials were systematically evaluated for their water oxidation activity under simulated light. Compared with the bare WO_3_, the N-CQD/*meso*-WO_3_ hybrid exhibited about 2.23 times higher oxygen evolution photocurrent density. The enhanced photocatalytic performance might be credited to the promoted photo-response range and highly efficient charge carrier separation.

## 2. Materials and Methods

### 2.1. Chemicals and Materials

Tungsten (VI), hexachloride (WCl_6_ ≥ 99.9%), and tetrahydrofuran (THF, ≥99.5%) were acquired from Sigma Aldrich, Missouri, USA. The precursors used for the CQDs and N-CQDs were sucrose (C_12_H_22_O_11_, ≥99.5%) and melamine (C_3_H_6_N_6_, ≥99%), respectively, which were obtained from Aldrich, Missouri, USA. Sodium sulfate (Na_2_SO_4_, ≥99%), isopropanol (C_3_H_8_O, ≥99.5%), and acetone (C_3_H_6_O, ≥99.5%) were purchased from BDH Middle East, Dubai, UAE. The fluorine-doped tin oxide was used as the substrate (FTO, resistivity = 10^−14^ Ω·cm^−2^, Lanbo Glass Ltd., Hong Kong, China). All reagents were used as received. Ultrapure water purification system of Milli-Q (Millipore, Inc., Darmstadt, Germany) was used to produce the deionized water with a resistivity of 18.2 MOhm cm. 

### 2.2. Preparation of CQDs, N-CQDs, and Mesoporous WO_3_ Hybrid Photoanodes

The N-doped CQDs were fabricated via a hydrothermal process using sucrose and melamine (C_3_H_6_N_6_) as the sources for carbon and nitrogen, respectively. In a typical synthesis, 7 wt. % of melamine (0.0565 g) was dispersed in 30 mL of deionized water under ambient conditions and agitated vigorously using magnetic stirring for 1 h. Afterward, 0.75 g of sucrose was introduced to the previous transparent mixed solution at ambient temperature and stirred continuously for a further 30 min. Subsequently, the obtained solution was kept in a 40 mL Teflon-lined autoclave reactor and subjected to thermal heating for 5 h at 180 °C. Later, the products were collected, washed with water/ethanol, and then subjected to the drying process in an oven at 60 °C. Finally, the produced N-CQDs samples were denoted as N-CQD-x, where x represented the weight percentage of melamine used in the synthesis process (3, 5, 7, 13, or 20 wt. %). The CQDs were prepared using a procedure similar to that mentioned above but without melamine. The *meso*porous WO_3_ (*meso*-WO_3_) thin films were synthesized according to a self-assembly surfactant template and dip-coating method following the procedure reported by Li et al. [23]. An amphiphilic di-block copolymer with the composition PEO117-b-PS198 (Mn = 25850 g·mol^−1^) was prepared using a previously reported procedure [23]. In a typical experiment, PEO117-b-PS198 (2.0 wt. %, 200 mg) was first dissolved in 10 g of THF, 0.6 g of concentrated HCl (37 wt. %) was carefully added slowly, and the subsequent mixture was magnetically stirred for 30 min. Afterward, WCl_6_ (0.6 g) was introduced to the template mixture and agitated continuously for 2 h, after which the template mixture became highly viscous. Subsequently, the FTO substrates were pre-cleaned with a water-detergent mixture, isopropanol, and acetone, respectively, and then rinsed with deionized water. The obtained sol-gel solution was coated onto the FTO substrates using a dip-coating method (Xdip-MV1, West Bengal, India) with a 300 mm·min^−1^ withdrawal speed to obtain a transparent thin-film substrate as displayed in Figure S5. Afterward, the obtained film was rested horizontally at 25 °C for 20 min, dried at 40 °C for 24 h, and then annealed at 100 °C for 24 h to form the WO_3_-surfactant templated films. Subsequently, the coating process was repeated until the desired number of layers (1–13 layers) of *meso*porous WO_3_ had been applied. Afterward, the as-deposited films on the FTO substrate were pyrolyzed using a temperature ramp of 1 °C·min^−1^ to reach a final temperature of 350 °C for 3 h under a nitrogen atmosphere to decompose the surfactant. The films were cooled to temperature naturally and then annealed at 450 °C for another 1 h in the open air to form the *meso*porous WO_3_ catalyst. To load different amounts of N-CQDs or CQDs, the *meso*-WO_3_ thin films were immersed in 25 mL (1.5 mg·mL^−1^) of a N-CQD or CQD solution for 1, 3, or 5 h. Lastly, the resulting N-CQD/or CQD/*meso*-WO_3_ hybrids were rinsed with water, subjected to drying process at 70 °C for 6 h, and stored in a desiccator for further characterization.

### 2.3. Material Characterization

The crystallography and phase data of the fabricated *meso*-WO_3_ samples were analyzed using powder X-ray diffraction (XRD, Rigaku Miniflex 600, Rigaku Corporation, Tokyo, Japan). The morphological structure of the fabricated *meso*-WO_3_ catalysts was observed using field emission scanning electron microscopy (FE-SEM, JSM-7000F JEOL system, Tokyo, Japan) armed with energy dispersion X-ray spectroscopy (EDS, INCA 400 Oxford, High Wycombe, UK). The *meso*porous and fine structure of the hybrids were further examined using high-resolution transmission electron microscopy (HRTEM, JEOL 2100 F, FEI Tecnai G2 F30, Tokyo, Japan). The samples were characterized using Fourier-transform infrared spectroscopy (FTIR, Perkin Elmer GX spectrophotometer, Ohio, USA) and X-ray photoelectron spectroscopy (XPS, Specs SAGE 150, Thermo Fisher Scientific, MA, USA), and room-temperature photoluminescence (PL) spectra were obtained using a spectrofluorophotometer (Perkin Elmer LS55, Ohio, USA). The optical natures of the fabricated catalysts were assessed using a UV-vis-NIR spectrophotometer (Shimadzu UV-2600, MD, USA). The thickness of the composite films was assessed using a Bruker Dektak XT profilemeter (Coventry, UK). Nitrogen adsorption isotherms were obtained by means of NOVA 2200e surface area analyzer (Quantachrome Instruments, Florida, USA).

### 2.4. Photoelectrochemical Measurements of the Hybrid Materials

The PEC characterizations were evaluated through the potentiostat (BioLogic SAS, VSP-0478, Paris, France) electrochemical system in a three-electrode glass cell with a quartz window in 0.5 M Na_2_SO_4_ electrolyte solution (pH ~6.8). The modified *meso*-WO_3_ deposited on the FTO photoanodes was employed as the working electrode, and the saturated calomel electrode (SCE) and Pt-mesh (0.5 × 1.0 cm^2^) were employed as the reference and counter electrode, correspondingly. The potential was normalized to the reference hydrogen electrode (RHE) using the equation, E_RHE_ = E_SCE_ + 0.244 V + 0.059 pH at 25 °C, where pH = 14 for 1.0 M KOH solution. Linear sweep voltammograms (LSV) were evaluated in the anodic direction in the dark and under illumination. A xenon lamp (300 W, HAL-320, JAPAN) provided with a solar spectrum of air mass 1.5 global (AM 1.5 G) irradiance. AM 1.5 G filter (was engaged as the light source. A Mott-Schottky (M-S) plot was acquired at a frequency of 1 kHz in the dark using an electrochemical impedance analyzer (BioLogic SAS, VSP-0478). Electrochemical impedance spectroscopy (EIS) analysis was performed in the dark and under illumination at an applied bias of 1.0 V vs. SCE.

## 3. Results and Discussion

### 3.1. Characterization of the CQDs and N-CQDs

**Figure 1:**
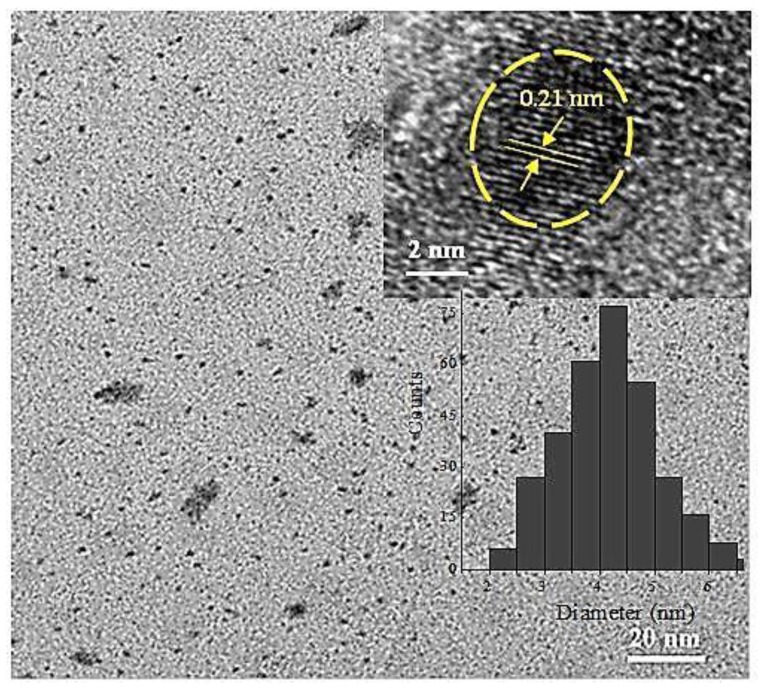
Transmission electron microscopy (TEM) image of the fabricated N-doped carbon quantum dots (7-N-CQDs), insets are the high-resolution TEM (HRTEM) image and its corresponding particle-size distribution histogram of 7-N-CQDs.

The size and microstructure of the as-prepared N-CQDs were investigated using transmission electron microscope (TEM). The TEM micrograph in Figure 1 shows that the N-CQDs were highly dispersed, with a size distribution of 2 to 7 nm and an average particle size of 4 nm, as shown in the inset particle size histogram. The HRTEM image of a single N-CQD (inset in Figure 1) clearly shows that the as-synthesized N-CQDs exhibited a lattice spacing of 0.21 nm, which corresponds to the d-spacing of the (100) crystal plane of graphite [38]. The surface chemistry and functional groups of the fabricated CQDs and N-CQDs were investigated using FTIR spectroscopy. The FTIR spectra, displayed in Figure S1, reveal that all the CQD and N-CQD samples exhibited a broad peak in the range 3100–3500 cm^−1^, which showed the existence of O–H and NH_2_ stretching vibrations [38,39]. The peaks at around 2925 cm^−1^ were credited to the CH_3_ and CH_2_ stretching modes, while the peak at 1705 cm^−1^ was ascribed to C=O stretching vibrations, likely associated with the existence of carboxylic acid or amide groups [38,40]. The peaks at 1616 cm^−1^ were assigned to the N-H bending vibration, while the peak at 1445 cm^−1^ corresponded to C–N vibrations [40]. Moreover, the bending vibrations of the C–O bond and the stretching vibrations peak of the C–O–C bond were observed at 1388 and 1027 cm^−1^, respectively [41]. Interestingly, as the nitrogen precursor (melamine) content was increased from 7 to 20%, the intensity of the N–H and C–N peaks at 1616 cm^−1^ and 1445 cm^−1^ was enhanced, while the opposite trend was observed for the C=O peak at 1705 cm^−1^. This suggests that as the nitrogen content of the N-CQDs was increased, a concurrent decrease in the oxygen content was observed.

The XPS study was carried out to assess the surface states and composition of the CQD and N-CQD samples. As shown in Figure S2a and by comparison to the CQD spectrum, the XPS survey spectrum for N-CQD-7 and N-CQD-20 contains C, N, and O peaks at binding energies of 285, 400, and 531 eV, respectively. The XPS spectra revealed that the elemental composition of N-CQD-20 was carbon (52.3%), nitrogen (20.97%), and oxygen (26.73%). These values were very close to those obtained from the EDX elemental analysis displayed in Figure S3. The high-resolution C 1s spectrum of the N-CQD sample displayed in Figure S2b exhibits four main peaks. The peak noticed at 284.05 eV was attributed to the C–C bonds of the graphitic structure, suggesting that the graphite structure predominated in the N-CQD sample. The peaks at 286.88, 290.23, and 293.24 eV can be allocated to carbon in the form of C–N, C–O (sp3), and C=O, correspondingly [42]. Deconvolution of the N 1s core spectrum in Figure S2c revealed two main peaks centered at 398.77 and 401.98 eV, confirming the presence of nitrogen atoms in pyrrolic- and graphitic-like C–N–C (sp3) and (C)3–N (sp3) structures, correspondingly [42,43]. The O 1s core spectrum of N-CQD-7 in Figure S2d might be deconvoluted into two main peaks at 530.1 eV and 532.80 eV, indicating the existence of C=O and C–OH or C–O–C, respectively [43]. The FTIR and XPS results indicate that the surfaces of the fabricated N-CQDs were functionalized with multiple oxygen and nitrogen groups. More importantly, the data described above confirmed that melamine successfully served as a nitrogen source during the hydrothermal synthetic route of the N-CQDs.

The optical nature of the CQD and N-CQD samples was examined using UV-Vis absorbance and PL spectroscopy as illustrated in Figure S4. UV-Vis spectra of the CQDs and N-CQDs dispersed in water are displayed in Figure S4a. The N-CQDs exhibited a strong UV-vis absorption peak at around 335 nm. This peak originated from the aromatic n-π* transition of the C=O, C–N, or –C–OH bonds, which may be from hydroxyl (–COOH) or amine (–NH_2_) groups on the surface of the N-CQDs [44]. The inset in Figure S4a shows that the N-CQD dispersion was transparent with a faint yellow color, while under a UV excitation wavelength of 365 nm, it exhibited blue fluorescence, demonstrating the bright luminescence of the prepared N-CQDs. This observation clearly indicated that the N-CQDs had different UV absorption and fluorescence emission behavior than the undoped CQDs. The PL emission spectra of the CQDs, N-CQD-7, and N-CQD-20 in the excitation wavelength range from 340 nm to 540 nm, as displayed in Figure S4b–d, respectively. The spectrum of the diluted N-CQD dispersion was dependent on the excitation wavelength, and the emission peaks varied from 440 to 525 nm under an excitation of 350–540 nm. Furthermore, not only was the fluorescence emission of the prepared N-CQDs stronger than that of the CQD solution of the same concentration, but the maximum fluorescence emission wavelength (λ_em_) was also blue-shifted, as displayed in Figure S4c. The fluorescence emission intensity of N-CQD-7 was 9.7 times stronger than that of CQD, which could be explained by a reduction in the number of non-radiative recombination centers on the N-CQD surface [44]. Interestingly, the PL intensity of the N-CQDs under UV excitation improved considerably as the N doping level was increased, as shown in Figure S4e. The N-CQDs with 7% N displayed the uppermost PL intensity among all the samples. In particular, the strongest emission band was positioned at 533.4 nm under 480 nm excitation. Further, the PL emission of the N-CQDs shifted to higher wavelength as its intensity increased. This behavior was attributed to the surface defect emission derived from the CQDs and was inconsistent with previous reports in the literature [45]. The external quantum yields (EQY) of the N-CQDs under 360 nm excitation were assessed using quinine sulfate (QS) as the calibration standard sample. As displayed in Figure S4e, N-CQD-7 revealed a greater EQY of 74.3%, which was superior to that of N-CQD-20 (60.4%), N-CQD−13 (33.2%), N-CQD-5 (18.4%), and N-CQD-3 (13.4%). This high PL EQY was mostly due to the attendance of O and N atoms. In particular, the heteroatoms in the CQD lattice could reduce the π-connections between the sp^2^-domains of carbon atoms, changing their electronic structure and thus suppressing non-radiative energy dissipation [42]. Significantly, increasing the N-doping level in the N-CQDs increased the photoexcitation efficiency, that is, fewer photo-induced electrons in the N-CQDs were quenched by energy traps. Thus, among the various N-CQDs studied, N-CQD-7 exhibited the best photosensitization performance for PEC applications. Thus, CQDs and N-CQD-7 were combined with the *meso*-WO_3_ photoanodes as photosensitizers for PEC water oxidation.

### 3.2. Characterization of Meso-WO_3_ and the CQD/Meso-WO_3_ and N-CQD/Meso-WO_3_ Hybrids

**Figure 2:**
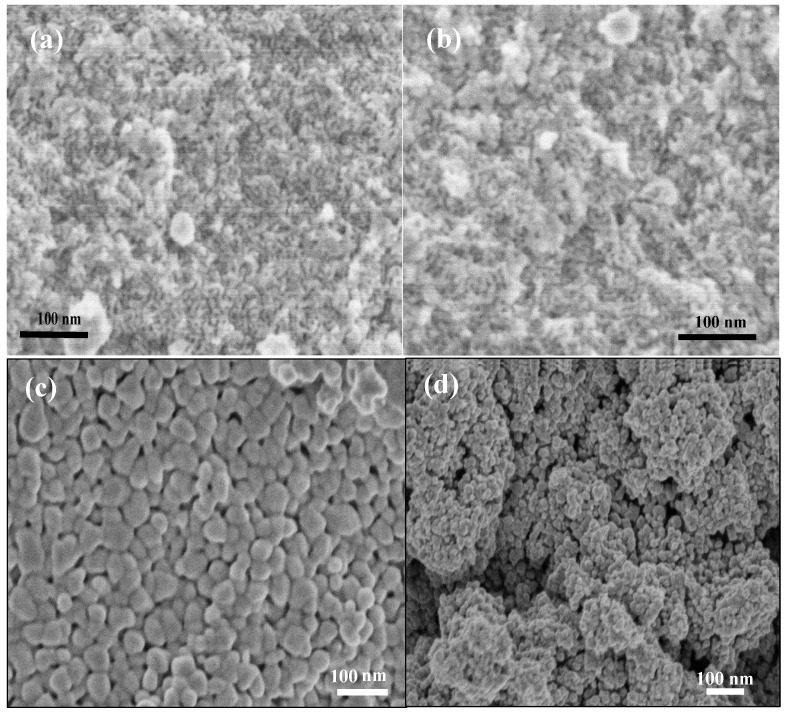
Field emission scanning electron (FESEM) images of (**a**) *meso*-WO_3_ catalyst after annealed at 350 °C in the N_2_ atmosphere for 3 h (**b**) *meso*-WO_3_ catalyst after the second step of annealing in air at 450 °C for 1 h. (**c**) WO_3_ annealed in one step at 450 °C in air (**d**) *bulk*-WO_3_ prepared under similar condition but in absence of PEO-PS template.

The morphology and *meso*structure of the *meso*porous tungsten trioxide modified with N-CQDs were examined using field emission scanning electron microscopy (FESEM) and HRTEM. The FESEM images of the attained *meso*-WO_3_ electrodes are displayed in Figure 2. In particular, the *meso*-WO_3_ catalyst annealed at 350 °C in N_2_ for 3 h (see Figure 2a) and then at 450 °C in air for 1 h (see Figure 2b) clearly exhibited a well-defined and interconnected *meso*porous framework that extended over a very large domain. The FESEM images revealed that the *meso*porous morphology of the catalyst did not change during annealing in N_2_ and air, which confirmed the successful creation of the WO_3_ *meso*porous structure. This can be explained by the fact that the residual carbon can maintain the tungsten oxide *meso*structures and prevent wall expansion during annealing in air after burning the surfactant in an N_2_ atmosphere. 

The residual carbon was then removed after annealing in air, leaving the high-surface-area WO_3_ *meso*porous structure. However, as shown in Figure 2c, direct one-step annealing in air at 450 °C for 2 h led to the destruction of the *meso*structures and the formation of adjoining WO_3_ particles with a mean particle size of 40 nm. One-step direct calcination in the air caused the concurrent removal of carbon and WO_3_ nanoparticle growth, which destroyed the *meso*porous structure. The FESEM image of the WO_3_, fabricated in the absence of a surfactant (*bulk*-WO_3_) in Figure 2d, does not exhibit any obvious pores, and the surface morphology of the *bulk*-WO_3_ consisted of an aggregated nanoparticle structure with a mean particle size of 20 nm. It worth noting that the surface area of the *bulk*-WO_3_ was 18 m^2^·g^−1^, which was significantly less than that of *meso*-WO_3_, as confirmed by the sorption isotherm results shown below.

**Figure 3:**
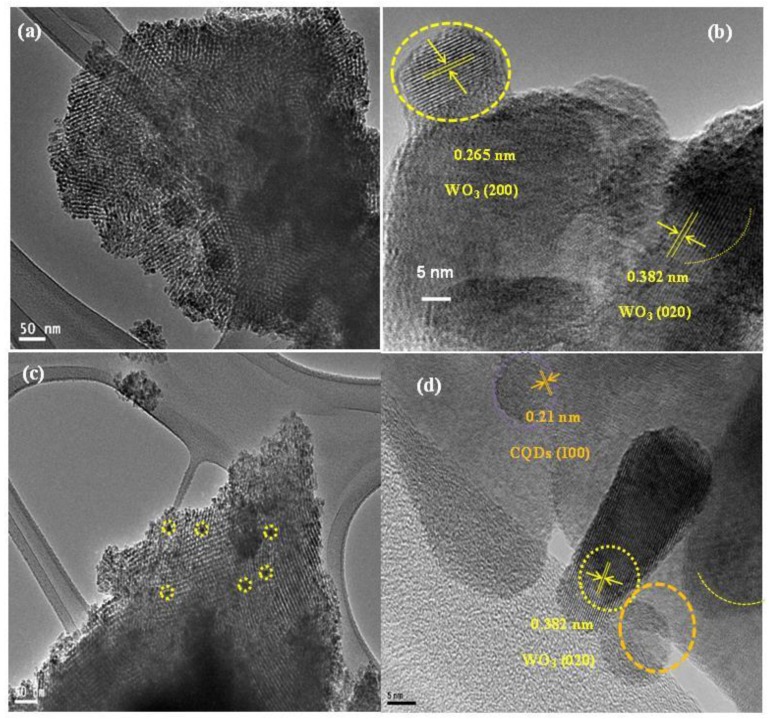
HRTEM images of (**a**) *meso*-WO_3_ (**b**) 7-N-CQDs/*meso*-WO_3_ sample, and a higher magnified HRTEM images of (**c**) *meso*-WO_3_, (**d**) 7-N-CQDs/*meso*-WO_3_ after annealing at 350 °C in N_2_ and 450 °C in air.

Figure 3 displays the HRTEM images of the *meso*-WO_3_ and the modified N-CQD-7/*meso*-WO_3_ catalysts. The HRTEM image displayed in Figure 3a reveals that the fine structure of the *meso*-WO_3_ catalyst exhibited distinct *meso*pores arranged in a highly ordered 2D hexagonal *meso*structure, with an average pore size of ~5 nm. Furthermore, the higher resolution TEM image in Figure 3b displays that the *meso*-WO_3_ framework consisted of well-crystallized walls with a lattice spacing of 0.265 nm and 0.382 nm, corresponding to the (200) and (020) diffraction planes of WO_3_ (JCPDS # 43–1305).

**Figure 4:**
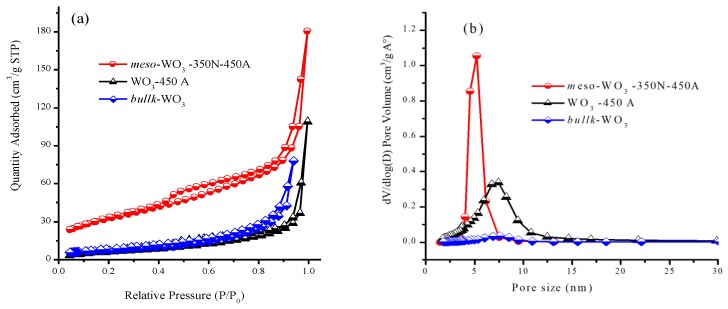
(**a**) N2 sorption isotherms and (**b**) Barrett, Joyner, and Halenda (BJH) pore size distributions of *meso*-WO_3_ and *bulk*-WO_3_ samples.

Figure 3c shows the TEM image of the modified N-CQD-7/*meso*-WO_3_ catalyst. The N-CQDs (dark spots) were attached to the surface of *meso*-WO_3_ (yellow circles) and the *meso*structures of the *meso*-WO_3_ substrate remained unchanged, demonstrating the successful loading of the N-CQDs on the surface of *meso*-WO_3_. The HRTEM image in Figure 3d evidently shows the (100) and (020) lattice fringes with a spacing of 0.21 and 0.382 nm, agreeing to the N-CQDs and WO_3_, respectively. Figure 4a reveals the N_2_ sorption isotherms of the *meso*-WO_3_ electrodes annealed under different annealing conditions, which exhibited type-IV curves with an H_2_-type hysteresis loop. For the *meso*-WO_3_ sample, the distinct capillary condensation step at a relative pressure of P/Po ~0.45 to 0.80 indicated the uniform *meso*porosity of the material. Another sharp N_2_ uptake with an H_2_-hysteresis loop at a higher relative pressure of P/P0 > 0.85 reflected the structural defects formed via pyrolysis. In contrast, the *bulk*-WO_3_ showed typical sorption isotherms resulting from the aggregation of the nanoparticles. Figure 4b shows the pore size distributions of the *meso*-WO_3_ electrodes calculated from the adsorption branch via Barrett-Joyner-Halenda (BJH) method, in which a uniform pore size of around 5 nm was observed. The detail of the textural properties of the *meso*-WO_3_ and *bulk*-WO_3_ are reported in Table S1. Clearly, the calculated Brunauer-Emmett-Teller (BET) surface area of the *meso*-WO_3_ was 105 m^2^·g^−1^, which was higher than that obtained for *bulk*-WO_3_ (18 m^2^·g^−1^) and *meso*-WO_3_ directly annealed in air at 450 °C (28 m^2^·g^−1^). Moreover, the high surface area, open porous structure, and uniform pore size of the *meso*-WO_3_ materials can be exploited for loading co-catalysts onto their surfaces, making them an ideal electrode material for various energy-related applications such as water splitting devices, solar cells, and batteries.

**Figure 5:**
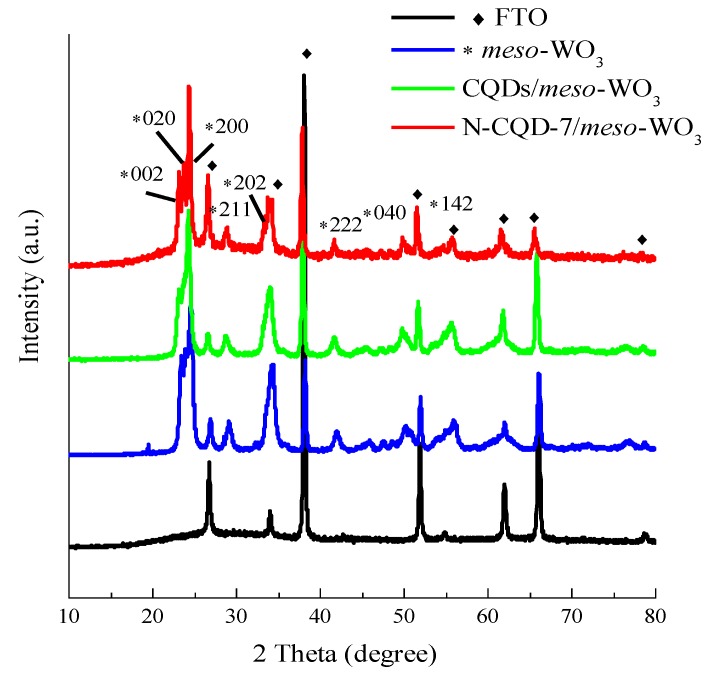
XRD patterns of *meso*-WO_3_, CQDs/*meso*-WO_3_ and N-doped CQDs/*meso*-WO_3_ composites.

As displayed in Figure S5, the *meso*-WO_3_ photoanodes were modified with CQD and N-CQD by dipping the *meso*-WO_3_/FTO photoanodes in the CQD solutions for different lengths of time after the annealing steps. The *meso*-WO_3_/FTO showed a light red color after being dipped in the CQD solution for 5 h, indicating the successful deposition of the CQDs onto the surface of WO_3_ (Figure S5, SI). Figure 5 reveals the diffraction patterns of the pure *meso*-WO_3_, CQD/*meso*-WO_3_, and N-CQD/*meso*-WO_3_ photoanodes. The diffraction peaks of pure *meso*-WO_3_ might be well-indexed to the planes of monoclinic WO_3_ (JCPDS No.01-072-0677), confirming the successful synthesis of *meso*-WO_3_. The XRD peaks of CQD/*meso*-WO_3_ and N-CQD/*meso*-WO_3_ were consistent with those of pure *meso*-WO_3_, indicating that the addition of the N-CQDs did not alter the crystalline phase of *meso*-WO_3_. The XRD peaks of the CQDs and N-CQDs could not be resolved in the CQD/*meso*-WO_3_ and N-CQD/*meso*-WO_3_ composites, possibly due to the low loading amount and dispersion of the CQDs, which was consistent with the literature [42].

**Figure 6:**
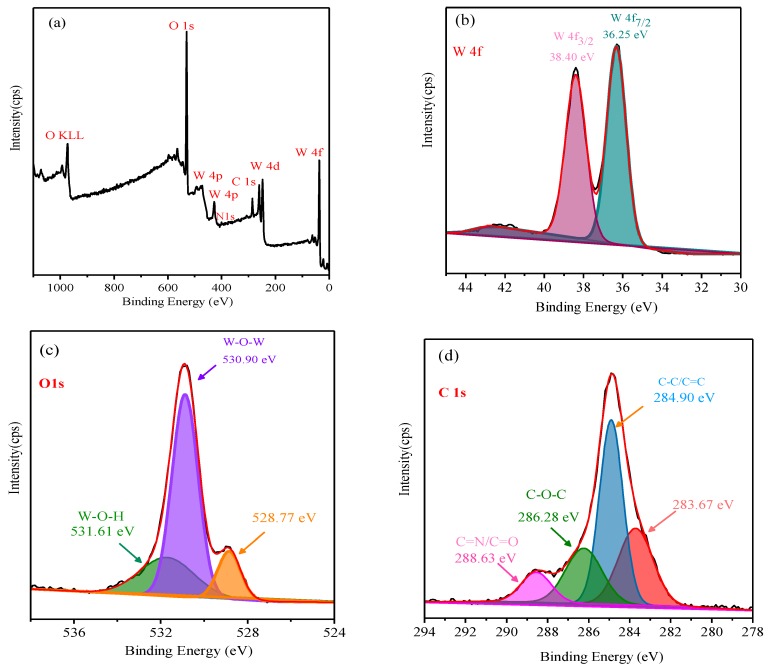

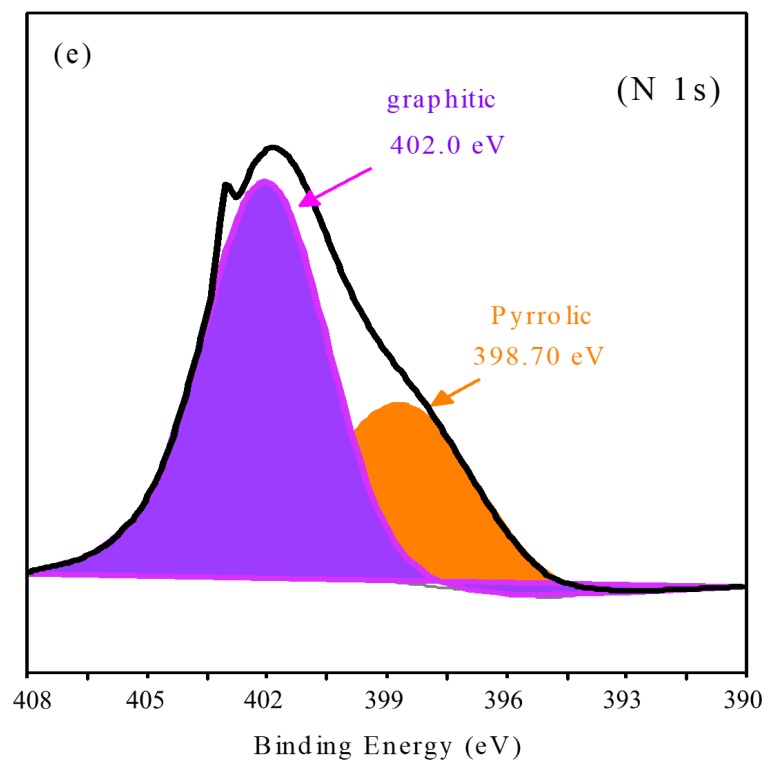
XPS spectra of as-synthesized N-CQDs/*meso*-WO_3_ composite, (**a**) survey, (**b**) W 4f, (**c**) O 1S, (**d**) C 1s, and (**e**) N 1s core spectrum.

The N-CQD-7/*meso*-WO_3_ electrode was examined using XPS analysis as displayed in Figure 6. The XPS wide survey spectrum of N-CQD/*meso*-WO_3_ in Figure 6a indicated the attendance of the elements W, O, C, and N at the surface of the photoanode. In Figure 6b, the core spectrum of W 4f shows two well-resolved peaks at 36.25 and 38.40 eV, which were assigned to the W 4f_7/2_ and W 2f_3/2_ orbital signals. Moreover, the narrow O 1s peak in Figure 6c can be deconvoluted to three peaks appearing at binding energies of 528.77, 530.90, and 531.61 eV, corresponding to the lattice oxygen of the layer-structured WO_3_ and adsorbed H_2_O or surface hydroxyl oxygen, correspondingly [15]. The C 1s high-resolution spectrum in Figure 6d contains three different contributions at 284.90, 286.28, and 288.63 eV, which were related to carbon in the C–C, C–O, and C=N/C=O states, respectively [15,16,17,18,19,20,21,22,23,24,25,26,27,28,29,30,31,32,33,34,35,36,37,38,39,40,41,42]. The high-resolution N 1s spectrum in Figure 6e exhibits two peaks centered at 398.70 and 402.0 eV, which were credited to the C–N–C and (C)_3_–N bonds [43,44], confirming that the surface of the N-CQD/*meso*-WO_3_ was rich in nitrogen-containing functional groups. Thus, the C 1s, O 1s, and N 1s spectra further demonstrated that the N-CQDs were loaded to the surface of the *meso*-WO_3_ substrate and that the N-CQD/*meso*-WO_3_ composites had successfully been fabricated.

**Figure 7:**
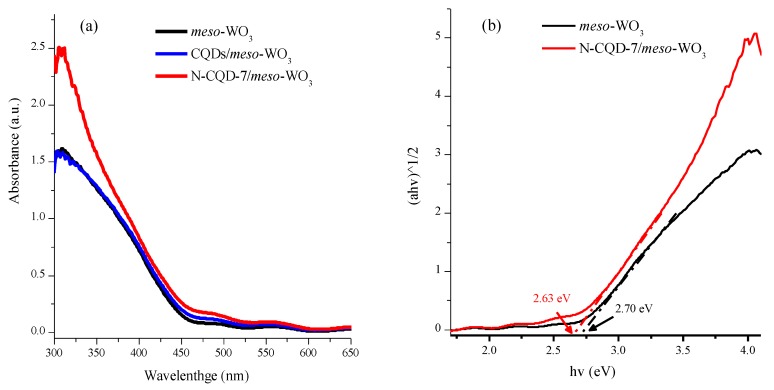
(**a**) UV-vis absorption of pristine *meso*-WO_3_, CQDs/*meso*-WO_3_, and N-CQDs/*meso*-WO_3_ photoanodes; (**b**) Tauc plots of the *meso*-WO_3_, and N-CQDs/*meso*-WO_3_ photoanodes.

To examine the effect of the CQD and N-CQD doping on the light-harvesting capability of *meso*-WO_3_, the optical properties of *meso*-WO_3_ and the CQD and N-CQD/*meso*-WO_3_ photoanodes were investigated using UV–vis absorption. As depicted in Figure 7a, the *meso*-WO_3_ photoanodes exhibited optical absorption in the range of 300–473 nm, and the absorption spectrum capacity in the visible-light region was significantly enriched by the addition of N-CQDs. Figure 7b displays the Tauc plots of the *meso*-WO_3_ and N-CQD/*meso*-WO_3_ composite films. The band gaps of the composite films were evaluated by plotting (αhν)1/2 against the photon energy [46]. The plot of (αhν)1/2 vs. the photon energy (hν), displayed in Figure 7b, was used to evaluate the bandgap energies of the *meso*-WO3 and N-CQD-7/*meso*-WO_3_ photoanodes. The bandgap of the pristine *meso*-WO_3_ photoanode was around 2.70 eV, while that of the N-CQD-7/*meso*-WO_3_ photoanode was 2.63 eV. Moreover, the conduction band (CB) and valence band (VB) of the *meso*-WO_3_ and N-CQD-7/*meso*-WO_3_ photoanodes were assessed using the following equation [47]:

E_CB_ = χ − E_e_ + 0.5 E_g_; E_VB_ = E_CB_ + E_g_ (1)

where χ represents the absolute electronegativity of the semiconductor, with the χ value of WO_3_ being 6.59 eV [48]; E_e_ is the energy of free electrons on the hydrogen scale (about 4.5 eV). Thus, the E_CB_ and E_VB_ of WO_3_ were assessed to be +0.74 eV and +3.44 eV for *meso*-WO_3_ respectively.

### 3.3. Photo-Electrochemical Properties of the Meso-WO_3_ Photoanodes

**Figure 8:**
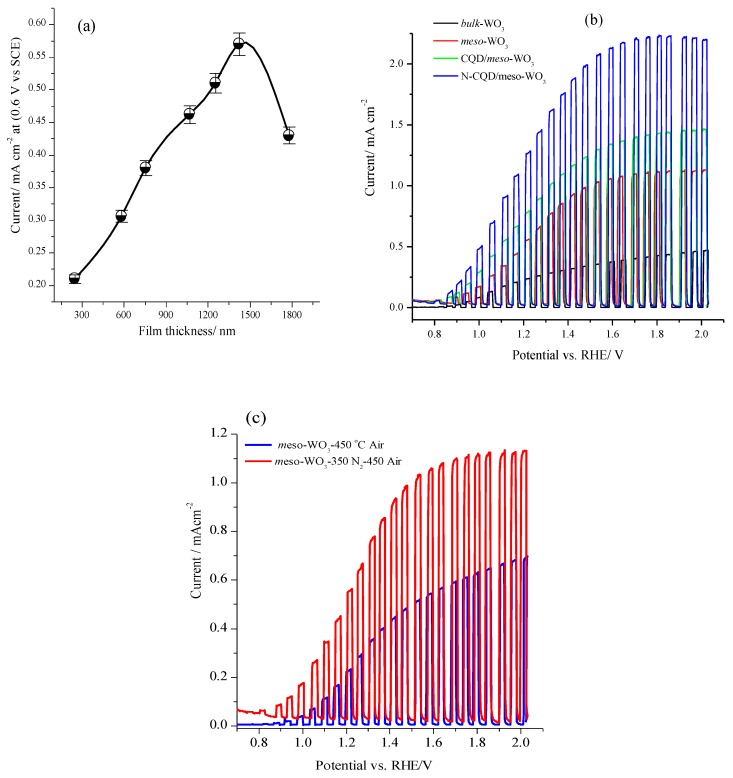
(**a**) Plot for the photocurrent obtained (at 1.23 V vs. RHE) using *meso*-WO_3_ photoanodes fabricated with various thicknesses, (**b**) chopped cyclic voltammograms for *meso*-WO_3_, CQDs/*meso*-WO_3_, and N-CQDs/*meso*-WO_3_ in 0.5 M Na_2_SO_4_ under visible-light excitations, (**c**) chopped cyclic voltammograms in 0.5 M Na_2_SO_4_ under irradiation for *meso*-WO_3_ photoanodes thermally annealed at 450 °C and 350 N_2_ and directly annealed in air at 450 °C upon visible illumination.

The PEC activities of the as-synthesized *meso*-WO_3_, CQD/*meso*-WO_3_, and N-CQD-7/*meso*-WO_3_ photoanodes toward water oxidation were investigated in the dark and under visible-light irradiation in a 0.5 M Na_2_SO_4_ solution (pH ~6.8) without adding a sacrificial agent or co-catalyst. Beforehand, the photocurrent of the *meso*-WO_3_ photoanode was optimized by varying the film thickness, which was measured by profilometer as displayed in Figure S6. Figure 8a displays the photocurrents obtained (at 1.23 V vs. RHE) using *meso*-WO_3_ photoanodes fabricated with various thicknesses as examined using chopped cyclic voltammograms under 100 mW·cm^−2^ visible-light excitation. As shown, the photocurrent increased from 0.21 to 0.58 mA·cm^−2^ as the thickness of the *meso*-WO_3_ film was increased from 250 nm to 1.42 µm due to promoted light absorption, and consequently, enhanced hole-electron pair photo-generation.

However, the photocurrent decreased when the thickness of the *meso*-WO_3_ reached about 1.78 µm due to an increase of the charge recombination rate. Thus, the optimum photocurrent of 0.58 mA·cm^−2^ was achieved at a film thickness of 1.42 µm and 1.23 V vs. RHE under standard conditions. These results are comparable with those previously published for *meso*porous WO_3_-based materials [7,49]. In addition to the film thickness, the surface film morphology and nanoarchitecture also influenced the PEC performance of the semiconductor photoanodes. Figure 8b shows the chopped LSVs of the *bulk*-WO_3_, *meso*-WO_3_, CQD/*meso*-WO_3_, and N-CQD-7/*meso*-WO_3_ photoanodes (film thickness about 1.4 µm) at 50 mV·s^−1^ under pulsed light irradiation in 0.5 M Na_2_SO_4_ solution (pH ~6.8). Upon illumination, the photocurrent of the photoanodes at 1.23 V vs. RHE decreased in the order N-CQD-7/*meso*-WO_3_ (1.45 mA·cm^−2^) > CQD/*meso*-WO_3_ (0.8 mA·cm^−2^) > *meso*-WO_3_ (0.65 mA·cm^−2^) > *bulk*-WO_3_ (0.25 mA·cm^−2^).

The *meso*-WO_3_ electrode exhibited a photocurrent density of 2.6 times greater than that of its corresponding *bulk*-WO_3_ electrode, which was credited to the greater number of active sites (higher surface area) of the *meso*-WO_3_ photoanodes exposed to the electrolyte, which enhanced the PEC activity. Furthermore, the N-CQD-7/*meso*-WO_3_ photoanodes demonstrated a significantly higher photocurrent density of 1.45 mA·cm^−2^, which was 1.8 and 2.23 times greater than that of the CQDs/*meso*-WO_3_ and bare *meso*-WO_3_, respectively. Therefore, the photocurrent properties of the pristine *meso*-WO_3_ were remarkably improved by modification with N-CQDs, which suggested that the N-doped CQDs dramatically improved the charge transfer between the CQDs and the *meso*-WO_3_ thin film via electron donation from the introduced N-atoms. As presented in Table S1, the photocurrent density of 1.45 mA·cm^−2^ at 1.23 V vs. RHE acquired for the N-CQD-7/*meso*-WO_3_ in our work was the highest recorded value for WO_3_ photoanodes decorated with carbon-based nanomaterials operating under similar conditions.

The influence of the direct annealing in air at 450 °C or in two-step annealing at 350 °C in N_2_ then at 450 °C in the air on the *meso*-WO_3_ photoanode PEC performance were investigated. Figure 8c shows the chopped LSVs at 50 mV·s^−1^ of *meso*-WO_3_ annealed under different conditions in a 0.5 M Na_2_SO_4_ solution and under pulsed light irradiation. Clearly, as shown in Figure 8c, the *meso*-WO_3_ photoanode annealed in two steps, firstly in N_2_ then in air exhibits an oxygen evolution potential onset potential that was approximately 100 mV lower under light illumination, as well as a higher photocurrent density of up to 0.68 mA·cm^−2^ at 1.23 V vs. RHE, which was nearly two times greater than that of the WO_3_ photoanode treated directly in air at 450 °C. This can be explained by the fact that annealing the WO_3_ in an N_2_ atmosphere first maintained the *meso*porous nature of WO_3_ due to the presence of the residual carbon after burning the surfactant, leading to a high surface area and more active sites, and thus higher PEC performance. On the other hand, direct annealing in air destroyed the *meso*porous WO_3_ framework, as confirmed by the SEM results in Figure 2c.

The corresponding applied bias photon-to-current efficiency (ABPE) of the *meso*-WO_3_, and N-CQD-7/*meso*-WO_3_ photoanodes under AM 1.5G illumination were estimated from the I–V curves via Equation (2).

(2) $$\mathrm{ABPE}\;\left(\%\right)=\left(\;\frac{\left[I\;\times \left[1.23\;v-V_{b\;}\right]\right]}{P_{tot}}\right)\;\times 100\%$$

where *I* = photocurrent density at applied bias *V_b_* (mA/cm^2^), *V_b_* = applied bias (V), and *P_tot_* = power density of the incident light (AM 1.5G, 100 mW·cm^−2^). In particular, the efficiency of the *meso*-WO_3_ photoelectrode at 1.0 V vs. RHE increased from 0.07% to 0.16% when the N-CQDs were incorporated into the *meso*porous structure. As mentioned earlier, the prolonged charge separation and transfer process of N-CQDs are the vital features for the promoted PEC behaviors of N-CQD/*meso*-WO_3_ photoanode.

**Figure 9:**
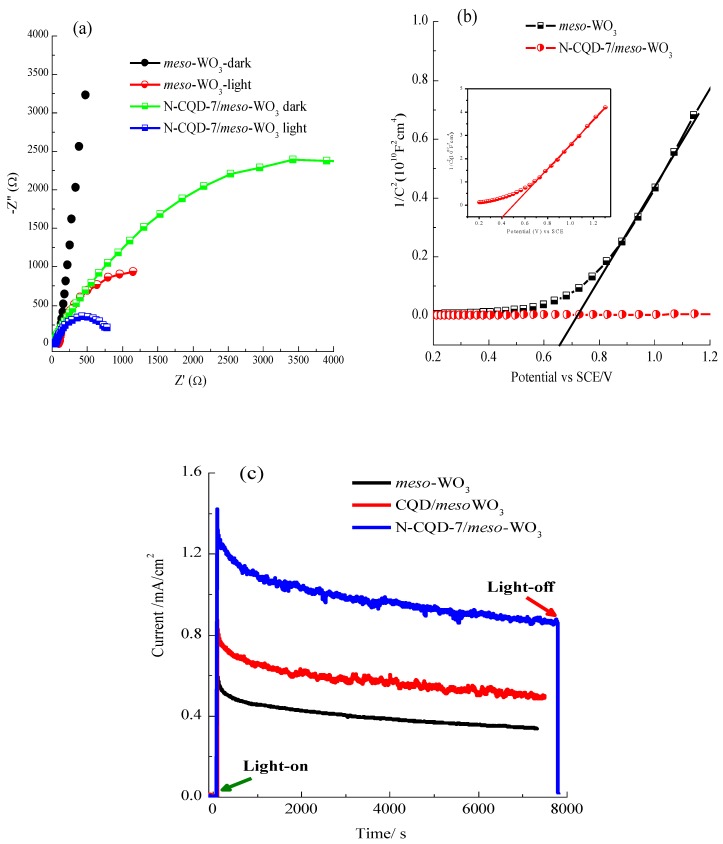
(**a**) Electrochemical impedance spectra (EIS) Nyquist-plots of pure *meso*-WO_3_ and 7-N-CQDs/*meso*-WO_3_ in the dark and under the irradiation conditions of AM 1.5G at a bias potential of 1.0 V vs. SCE with an AC voltage amplitude of 20 mV; (**b**) Mott–Schottky plots of the bare *meso*-WO_3_ and N-CQDs/*meso*-WO_3_ with a frequency of 1000 Hz in the dark and amplitude of 20 mV, (potential range 0.2–1.3 V); and (**c**) current-time curve of the *meso*-WO_3_, CQDs/*meso*-WO_3_, and 7-N-CQDs/*meso*-WO_3_ composite electrode at an applied bias of 0.6 V vs. SCE under the illumination of AM 1.5G in 0.5M Na_2_SO_4_ solution (pH 6.8), light intensity 100 mW/cm^2^.

To obtain a better realization of the interfacial charge transfer behavior in the *meso*porous modified WO_3_ photoanodes, an analysis of their EIS was performed. Figure 9a displays the Nyquist plots of the bare *meso*-WO_3_ and N-CQD-7/*meso*-WO_3_ electrodes at 1.23 V vs. RHE in the dark and under irradiation conditions.

The arc radius in the Nyquist plots indicates the charge transfer resistance at the electrode/electrolyte interface. The arc radius of the N-CQD-7/*meso*-WO_3_ electrode under both dark and light conditions was clearly smaller than those in the corresponding pristine *meso*-WO_3_ spectra, indicating faster interfacial charge transfer across the interface between the electrode and electrolyte and resulting in higher PEC performance [33,34,50]. In addition, the observed results evidenced that the N-doped CQDs increased the separation of charges (electrons and holes) in the N-CQD/*meso*-WO_3_ electrode, and thus contributed to its higher PEC activity. Figure 9b displays the Mott−Schottky (M-S) plots of the N-CQD/*meso*-WO_3_ and bare *meso*-WO_3_ electrodes. A positive slope was observed in the M–S plots of bare *meso*-WO_3_ and N-CQD-7/*meso*-WO_3_, as expected for n-type semiconductors. The M-S curves clearly demonstrated that the N-CQD-7/*meso*-WO_3_ electrode had a smaller slope than the pure *meso*-WO_3_ sample, indicating the increased donor density and conductivity of the former.

The donor density of the pristine *meso*-WO_3_ and N-CQD/*meso*-WO_3_ electrode were estimated from the slopes of the M-S curves. The calculated donor density of N-CQD-7/*meso*-WO_3_ was 2.30 × 1021 cm^−3^, which was higher than that of pristine *meso*-WO_3_ (4.01 × 1019 cm^−3^). The improved donor density was credited to the presence of nitrogen in N-CQD, which reduced the charge recombination of hole-electron pairs and was thus credited to the superior photocurrent behavior of the N-CQD-7/*meso*-WO_3_ photoanode. Furthermore, by extrapolating the linear M-S curve to the potential axis as presented in Figure 9b, the flat band potential (V_FB_) of the pure *meso*-WO_3_ and N-CQD/*meso*-WO_3_ electrodes were estimated to be 0.658 and 0.405 V vs. SCE, correspondingly. The V_FB_ of N-CQD/*meso*-WO_3_ was more negatively shifted and smaller than that of pure *meso*-WO_3_, which matched the cathodic shift of the oxygen evolution reaction overpotential. The obvious negative shift of V_FB_ in N-CQD/*meso*-WO_3_ enhanced the band bending at the interface of the N-CQD/*meso*-WO_3_ and electrolyte, thereby decreasing the recombination of the photoinduced charge-carriers and the overpotential in the oxygen evolution reaction (OER) kinetics of the N-CQD/*meso*-WO_3_ photoelectrode [51].

Finally, the durability of the *meso*-WO_3_, CQD/*meso*-WO_3_, and N-CQD-7/*meso*-WO_3_ photoanodes was investigated using chronoamperometry in an 0.5 M Na_2_SO_4_ solution at an applied voltage of 0.6 V vs. SCE under AM 1.5G illumination, as displayed in Figure 9c. The photocurrent-time profile of N-CQD-7/*meso*-WO_3_ exhibited better stability than that of bare *meso*-WO_3_, with 65.6% of its initial performance being maintained (0.88 vs. 1.34 mA·cm^−2^) after 2 h of testing. This demonstrated the role of the N-CQDs in improving the stability of the *meso*-WO_3_ by reducing the charge-carrier recombination process or by the rapid and complete OER reaction [52,53].

**Figure 10:**
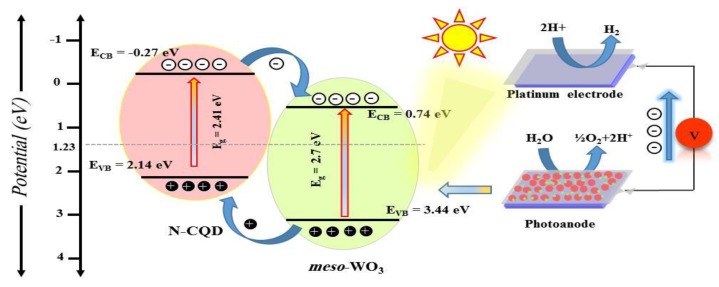
Proposed schematic energy-level diagram and charge migration process of N-CQDs/*meso*- WO_3_ system for the photo-electrochemical (PEC) water oxidation.

Figure 10 shows a proposed PEC water-splitting mechanism for the enhanced performance of the present N-CQD-7/*meso*-WO_3_ photoanodes. According to molecular orbitals (MOs) theory, various electronic transitions in N-CQD can be triggered the HOMO-LUMO (highest occupied molecular orbital, lowest unoccupied molecular orbital) energy levels under excitation of visible light [38,54], which results in a beneficial energy gap. Based on the linear potential scan method, the HOMO and LUMO positions of the N-CQD were assessed to be ≈−0.27 and 2.14 eV (Figure 10), correspondingly. Moreover, the bandgap energy of N-CQDs of the electron transition obtained from the potential scans was 2.41 eV, which is consistent with earlier reports [55]. Besides, on the basis of Butler and Ginley’s method, [56] the VB and CB energy levels of *meso*-WO_3_ were assessed to be 0.74 and 3.44 eV vs. RHE, respectively. In addition, considering that the HUMO potential of N-CQDs was more positive than its corresponding VB potential of *meso*-WO_3_, the photoexcited holes from VB of *meso*-WO_3_ can instinctively transfer to the HUMO of N-CQDs. Simultaneously, the photoexcited electrons can also be transferred from N-CQDs to *meso*-WO_3_. Hence, photoexcited electrons-holes were efficiently separated and the lifetime of the charge carriers was considerably continued. Lastly, the prolonging charge-carrier separation and transfer process can be credited to the enriched PEC response as argued earlier.

## 4. Conclusions

In summary, we productively synthesized a tungsten trioxide WO_3_ *meso*porous electrode via surfactant self-assembly and dip-coating methodologies using a high molecular-weight PEO-b-PS copolymer as a structure-directing template. This was followed by the incorporation of N-CQDs within the ordered *meso*structure through impregnation assembly. Importantly, the resulting N-CQD/*meso*-WO_3_ nanocomposites revealed considerably promoted PEC performance, with greater photocurrent densities (1.45 mA·cm^−2^ at 1.23 V vs. RHE) and low onset potentials (negatively shifted by 94 mV) compared to those of pristine *meso*-WO_3_. Furthermore, the N-CQD/*meso*-WO_3_ photoanode demonstrated a high ABPE of 0.16% at 1.0V vs. RHE. These results highlighted the multifunctional role of the hybridization of *meso*porous WO_3_ with N-doped CQDs in the enhancement of PEC solar water splitting.

## Supplementary Materials

The following are available online at https://www.mdpi.com/2079-4991/9/10/1502/s1, Figure S1: FTIR spectra of CQDs and N-CQDs samples, Figure S2: (a) XPS survey spectra for the CQDs, N-CQD-7 and N-CQD-20 samples. The only elements identified were carbon, nitrogen, and oxygen. (c) C 1s, (d) N 1s and (E) O 1s high resolution XPS spectra for N-CQD-7 sample, Figure S3: EDX spectra of N-CQDs samples, (a) N-CQD-3, (b) N-CQD-5, (c) N-CQD-7, and (d) N-CQD-20, Figure S4: (a) UV-Vis absorption spectra for the CQDs and N-CQDs samples. Inserts show digital photos of aqueous N-CQD-7 (left) and their bright blue PL (right) under UV, (b,c,d) PL spectra for the N-CQD-7, N-CQD−13, and N-CQD-20. The excitation wavelength was increased from 340 to 540 nm in 20 nm increments. (e) EQYs for the N-CQDs samples under 360 nm excitation, calibrated against quinine sulfate, Figure S5: steps for the synthesis of *meso*-WO_3_ and modification with CQDs, Figure S6: Thickness of *meso*-WO_3_ and modification with CQDs as measured by a profilometer, Table S1: a comparison the photocurrent density of WO_3_ based composite materials.
