# Supplementary material for: Mesoporous Tungsten Trioxide Photoanodes Modified with Nitrogen-Doped Carbon Quantum Dots for Enhanced Oxygen Evolution Photo-Reaction
Source: Nanomaterials (Basel). 2019 Oct 22;9(10):1502. doi: 10.3390/nano9101502 (PMC6836200; doi:10.3390/nano9101502)
Supplement: Supplementary file 1 [file nanomaterials-09-01502-s001.pdf]

nanomaterials

Article

Mesoporous Tungsten Trioxide Photoanodes Modified with Nitrogen-Doped Carbon Quantum Dots for Enhanced Oxygen Evolution Photo-Reaction

Mabrook S. Amer [1], Prabhakarn Arunachalam [1], Abdullah M. Al-Mayouf [1], Saradh Prasad [2], Matar N. Alshalwi [1] and Mohamed A. Ghanem [1,*]

[1] Electrochemical Sciences Research Chair (ESRC), Chemistry Department, College of Science, King Saud University, Riyadh 11451, Saudi Arabia; msamer@ksu.edu.sa (M.S.A.); parunachalam@KSU.EDU.SA (P.A.); amayouf@ksu.edu.sa (A.M.A.-M.); malshalwi@KSU.EDU.SA (M.N.A.)

[2] Department of Astronomy and Physics, College of Science, King Saud University, Riyadh 11451, Saudi Arabia; sprasad@KSU.EDU.SA

* Correspondence: mghanem@ksu.edu.sa; Tel.: +966-114670405

Supporting Information

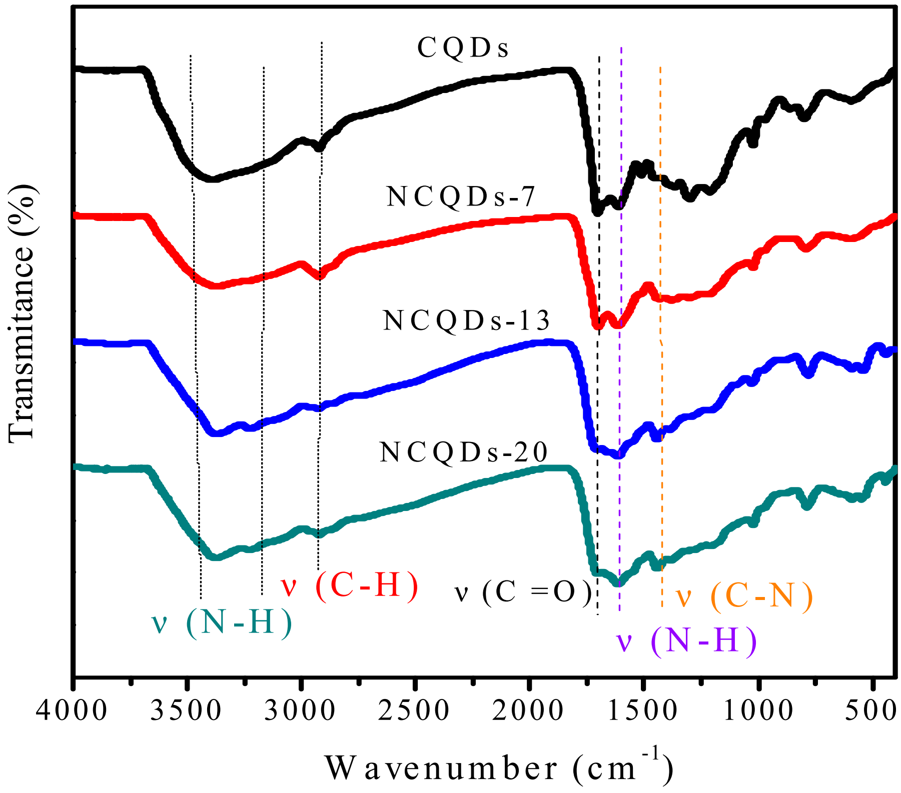

Figure 1. FTIR spectra of CQDs and N-CQDs samples.

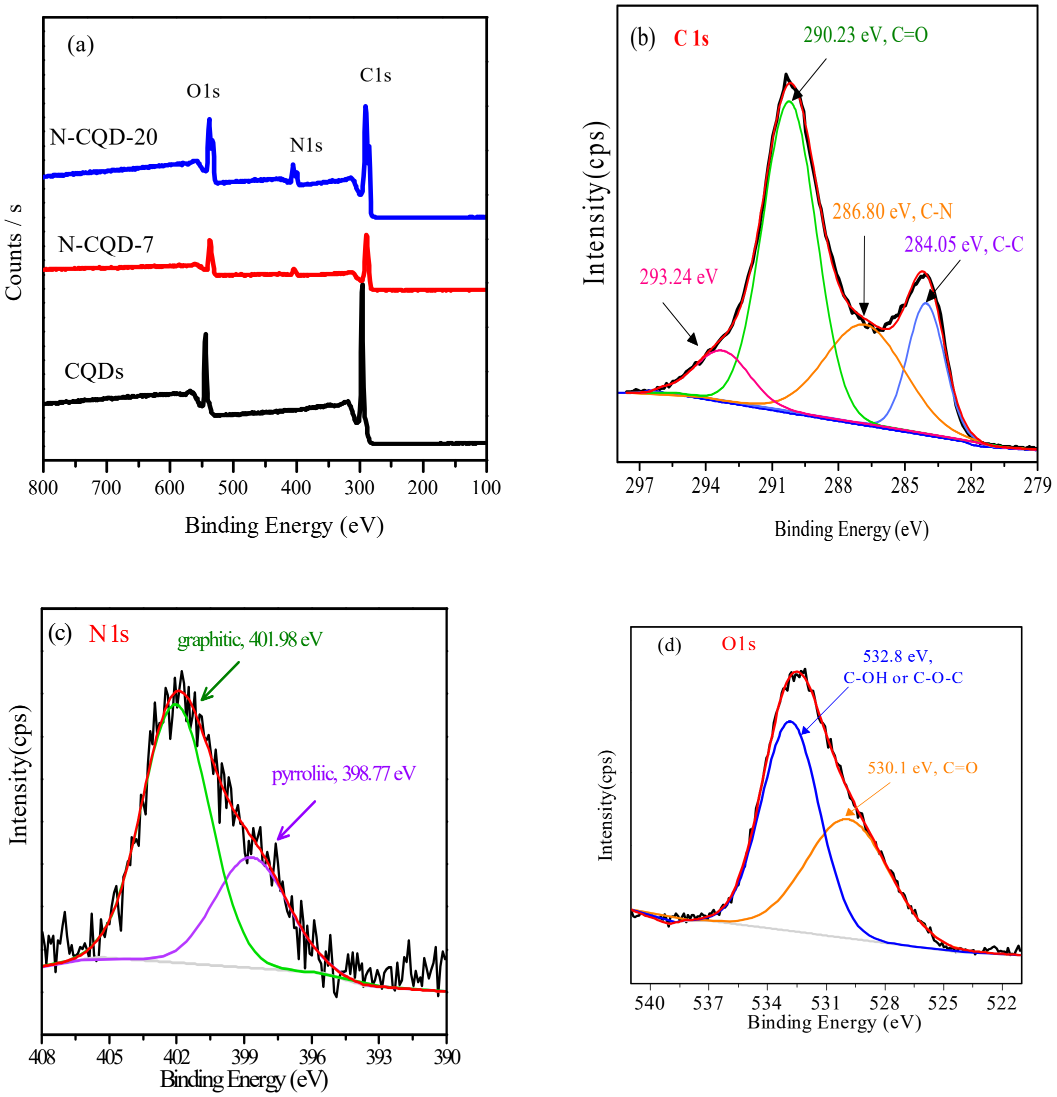

Figure 2. (a) XPS survey spectra for the CQDs, N-CQD-7 and N-CQD-20 samples. The only elements identified were carbon, nitrogen and oxygen. (c) C 1s, (d) N 1s and (E) O 1s high resolution XPS spectra for N-CQD-7 sample.

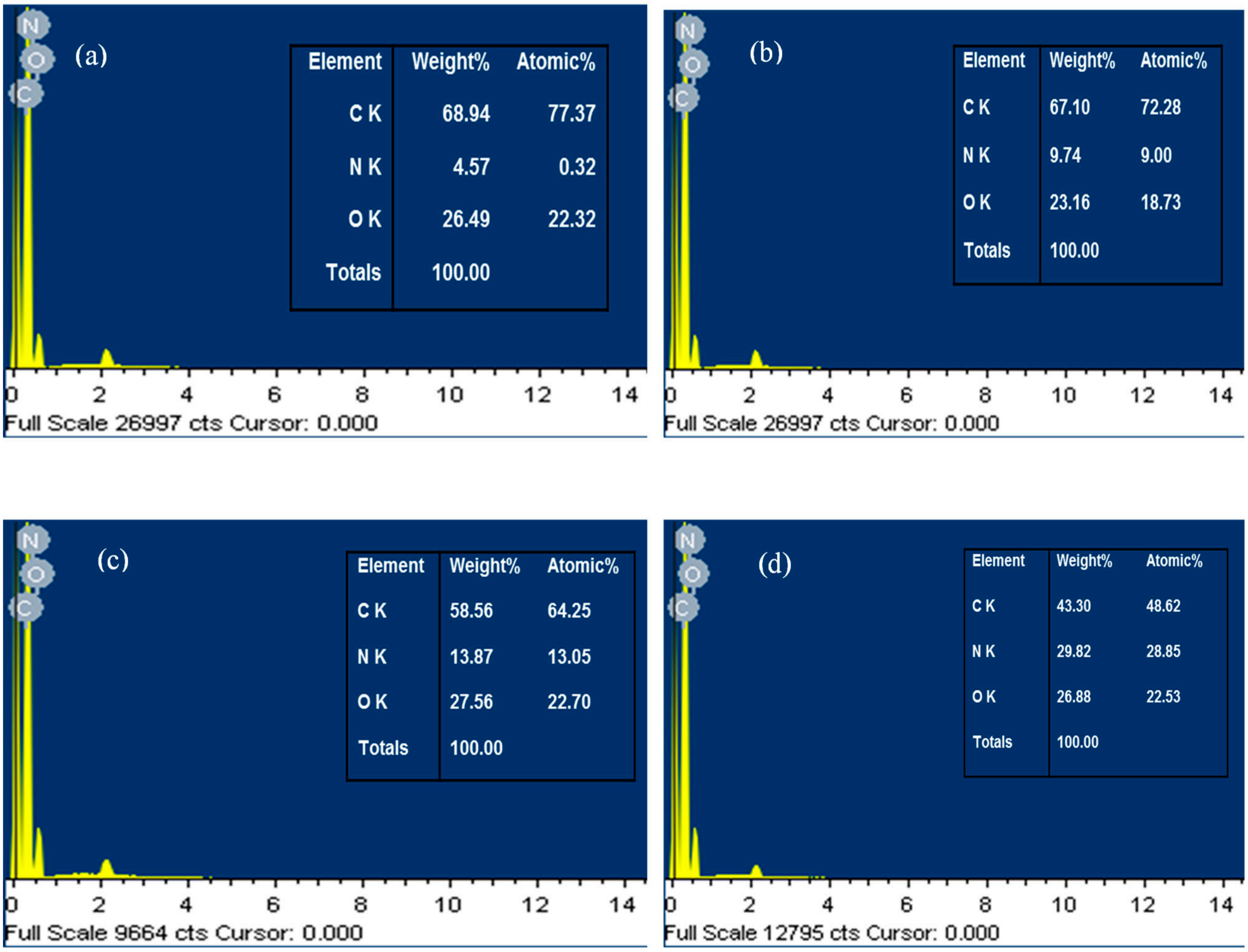

Figure S3. EDX spectra of N-CQDs samples, (a) N-CQD-3, (b) N-CQD-5, (c) N-CQD-7, and (d) N-CQD-20.

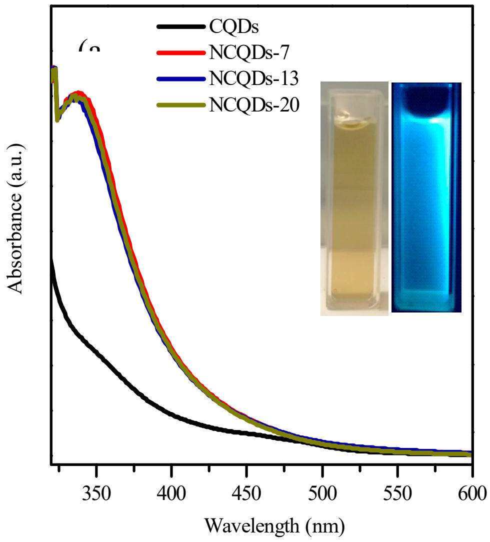

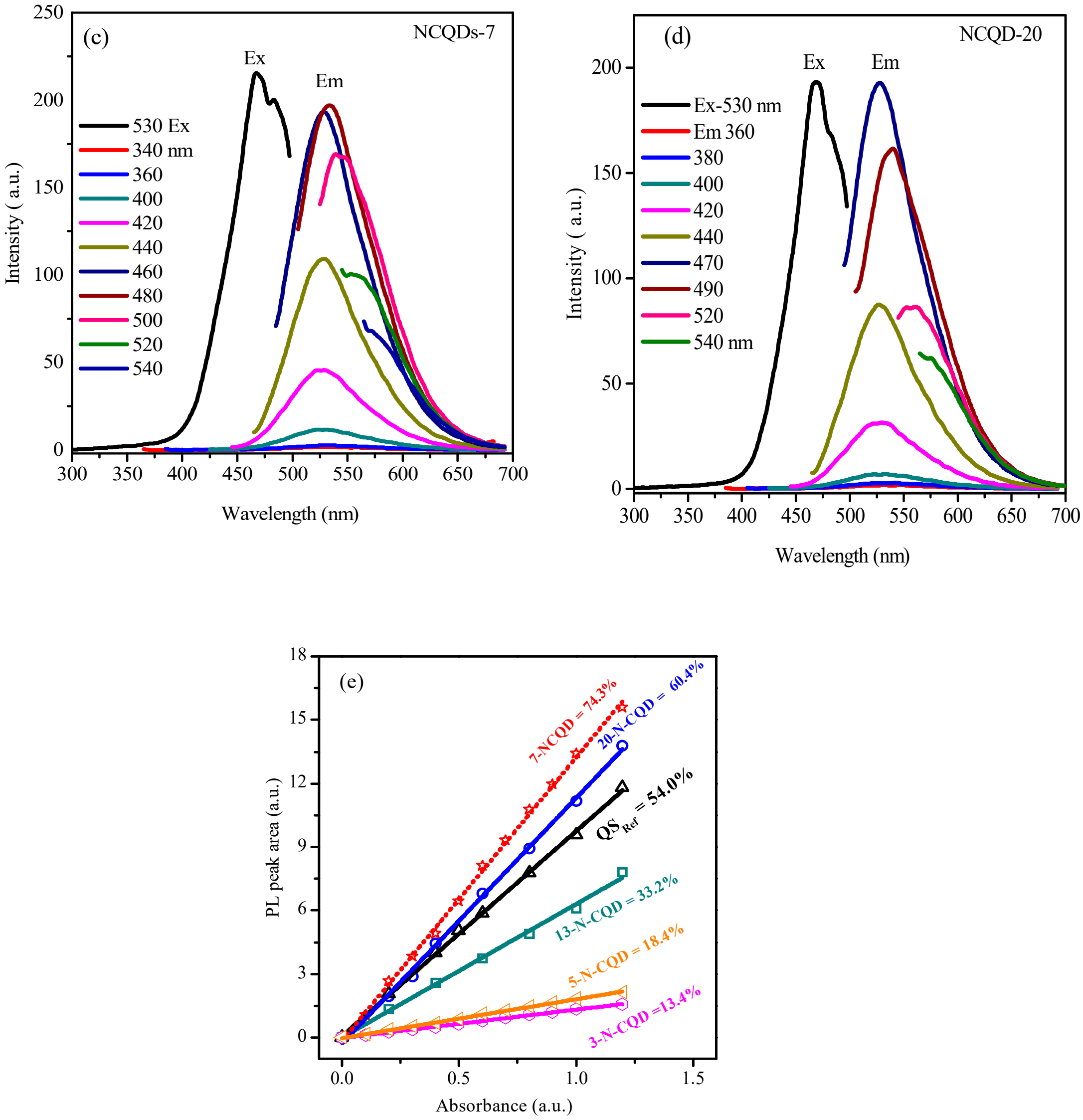

Figure S4. (a) UV-Vis absorption spectra for the CQDs and N-CQDs samples. Inserts show digital photos of aqueous N-CQD-7 (left) and their bright blue PL (right) under UV, (b,c,d) PL spectra for the N-CQD-7, N-CQD-13 and N-CQD-20. The excitation wavelength was increased from 340 to 540 nm in 20 nm increments. (e) External quantum yields for the N-CQDs samples under 360 nm excitation, calibrated against quinine sulfate.

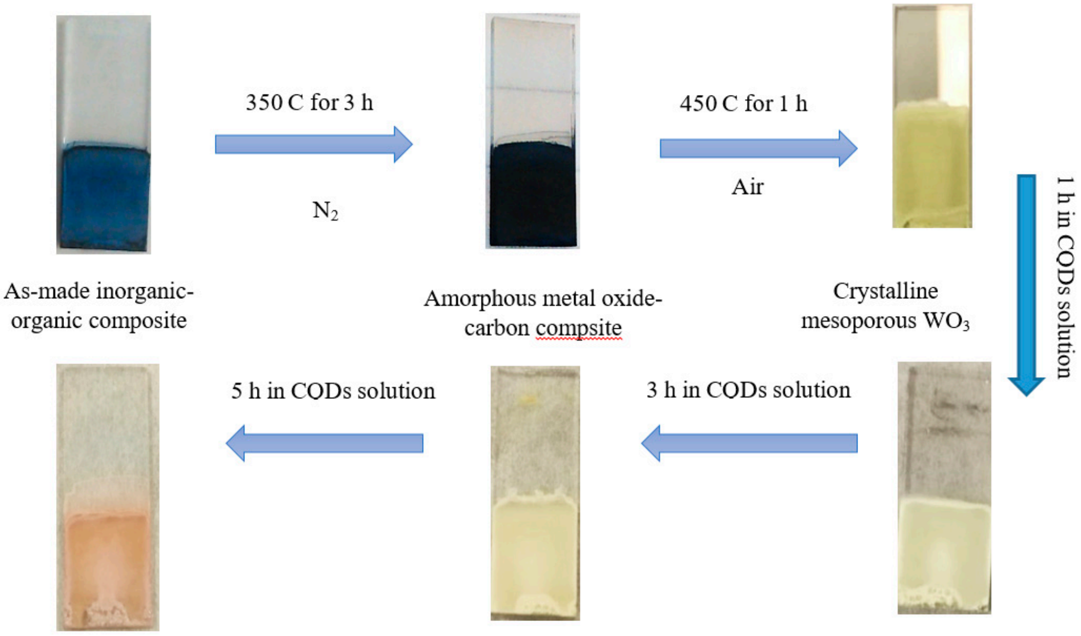

Figure 5. steps for the synthesis of *meso*-WO_3 and modification with CQDs.

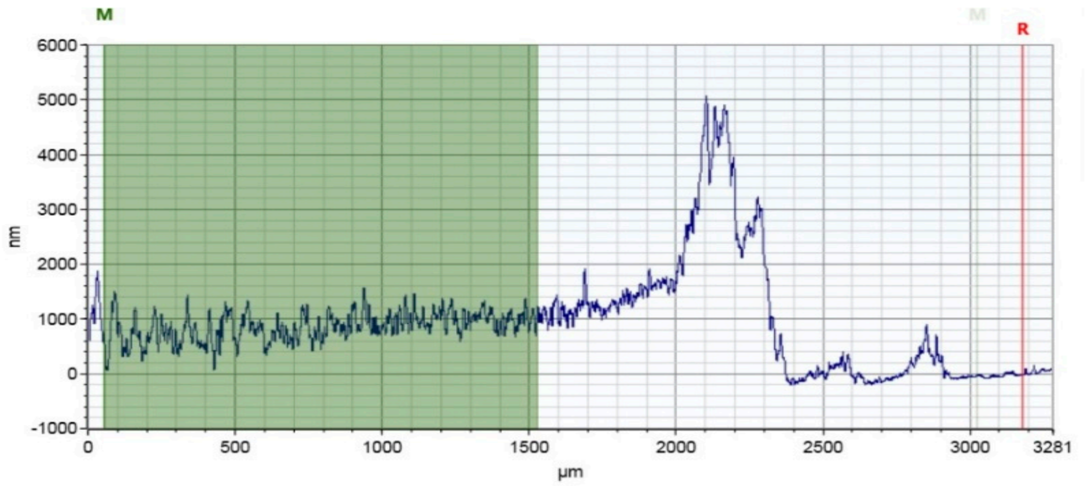

Figure 6. Thickness of of *meso*-WO_3 and modification with CQDs as measured by prophilometer.

Table 1. Comparison the photocurrent density of WO_3 based composite materials.

Photoelectrodes	Synthesis method	BET surface area (m²/g)	Pore volume cm³/g	Pore size (nm)	Photocurrent Density	Light Source	Electrolyte	Ref.
NCQDs/*meso*-WO_3	Impregnation/ surfactant self-assembly	105	0.27	5.0	1.45 mA cm^{-2}, 1.23 V vs RHE	AM 1.5G, 100 mW cm^{-2}	0.5 M Na_2SO_4	This Work
NCQDs/*meso*-WO_3 (450 °C)	Impregnation/ surfactant self-assembly	28	0.071	7.5	0.40 mA cm^{-2}, 1.23 V vs RHE	AM 1.5G, 100 mW cm^{-2}	0.5 M Na_2SO_4	This Work
Bulk-WO_3	Impregnation/ no surfactant	18	0.038	--	0.25 mA cm^{-2}, 1.23 V vs	AM 1.5G, 100 mW cm^{-2}	0.5 M Na_2SO_4	This Work

CQDs/WO_3 nanoplates	immersing /hydrothermal	--		--	1.18 mA cm^{-2}, 1.23 V vs RHE	simulated solar light	0.5 M Na_2SO_4	S1
CQDs/WO_3 nanoflakes	Seed-mediated solvothermal	--		--	1.46 mA cm^{-2}, 1.0 V vs. Ag/AgCl	AM 1.5G, 100 mW cm^{-2}	1 M H_2SO_4	S2
NCDs/WO_3 nanoflakes	Seed-mediated hydrothermal	--	--	--	1.42 mA cm^{-2}, 1.0 V vs. SCE	AM 1.5G, 100 mW cm^{-2}	1 M H_2SO_4	S3
CDots/WO_3 nanorods	Reflux/hydrothermal	--	--	--	11.5 µA cm^{-2}	150 W Xe lamp, (780> λ> 420 nm)	0.1 M Na_2SO_4	S4
Z-scheme WO_3/C_3N_4	Electrophoretic deposition	--	--	--	0.82 mA cm^{-2}, 1.23 vs RHE	AM 1.5G, 100 mW cm^{-2}	0.5 M Na_2SO_4	S5
Nanoporous carbon / WO_3	Wet chemistry	--	--	--	~ 8 µA cm^{-2}, 0.6 V vs. Ag/AgCl	blue LED, 371 nm	0.5 M Na_2SO_4	S6
rGO/WO_3	Thermal treatment	31.7	--	--	~1.1 mA cm^{-2}, 1 V vs Ag/AgCl	AM 1.5 G, 100 mW cm^{-2}	0.5 M H_2SO_4	S7
rGO/Nano-plate-WO_3	Wet chemistry / thermal decomposition	175.6	--	--	30 µA cm^{-2}, 0.6V vs. Ag/AgCl	AM 1.5G, 100 mW cm^{-2}	0.1 M Na_2SO_4	S8

References

1. Zhao, Z.; Butburee, T.; Peerakiatkhajohn, P.; Lyu, M.; Wang, S.; Wang, L.; Zheng, H. Carbon Quantum Dots sensitized Vertical WO_3 Nanoplates with Enhanced Photoelectrochemical Properties. *ChemistrySelect* **2016**, *1*, 2772–2777.
2. Shi, W.; Zhang, X.; Brillet, J.; Huang, D.; Li, M.; Wang, M.; Shen, Y. Significant enhancement of the photoelectrochemical activity of WO_3 nanoflakes by carbon quantum dots decoration. *Carbon N. Y.* **2016**, *105*, 387–393.
3. Kong, W.; Zhang, X.; Liu, S.; Zhou, Y.; Chang, B.; Zhang, S.; Fan, H.; Yang, B. N Doped Carbon Dot Modified WO_3 Nanoflakes for Efficient Photoelectrochemical Water Oxidation. *Adv. Mater. Interfaces* **2019**, *6*, 1–8.
4. Zhang, J.; Ma, Y.; Du, Y.; Jiang, H.; Zhou, D.; Dong, S. Carbon nanodots/WO_3 nanorods Z-scheme composites: Remarkably enhanced photocatalytic performance under broad spectrum. *Appl. Catal. B Environ.* **2017**, *209*, 253–264.
5. Wang, C.H.; Qin, D.D.; Shan, D.L.; Gu, J.; Yan, Y.; Chen, J.; Wang, Q.H.; He, C.H.; Li, Y.; Quan, J.J.; et al. Assembly of g-C_3N_4-based type II and Z-scheme heterojunction anodes with improved charge separation for photoelectrojunction water oxidation. *Phys. Chem. Chem. Phys.* **2017**, *19*, 4507–4515.
6. Gomis-Berenguer, A.; Celorrio, V.; Iniesta, J.; Fermin, D.J.; Ania, C.O. Nanoporous carbon/WO_3 anodes for an enhanced water photooxidation. *Carbon N. Y.* **2016**, *108*, 471–479.
7. Lin, J.; Hu, P.; Zhang, Y.; Fan, M.; He, Z.; Ngaw, C.K.; Loo, J.S.C.; Liao, D.; Tan, T.T.Y. Understanding the photoelectrochemical properties of a reduced graphene oxide-WO_3 heterojunction photoanode for efficient solar-light-driven overall water splitting. *RSC Adv.* **2013**, *3*, 9330–9336.
8. Prabhu, S.; Manikumar, S.; Cindrella, L.; Kwon, O.J. Charge transfer and intrinsic electronic properties of rGO-WO_3 nanostructures for efficient photoelectrochemical and photocatalytic applications. *Mater. Sci. Semicond. Process.* **2018**, *74*, 136–146.

© 2019 by the authors. Submitted for possible open access publication under the terms and conditions of the Creative Commons Attribution (CC BY) license (http://creativecommons.org/licenses/by/4.0/).
